# Chiral-induced unidirectional spin-to-charge conversion

**DOI:** 10.1126/sciadv.ado4285

**Published:** 2025-01-01

**Authors:** Ashish Moharana, Yael Kapon, Fabian Kammerbauer, David Anthofer, Shira Yochelis, Hadar Shema, Elad Gross, Mathias Kläui, Yossi Paltiel, Angela Wittmann

**Affiliations:** ^1^ Institute of Physics, Johannes Gutenberg University Mainz, Mainz 55128, Germany.; ^2^ Institute of Applied Physics, The Hebrew University of Jerusalem, Jerusalem 9190401, Israel.; ^3^ Center for Nanoscience and Nanotechnology, The Hebrew University of Jerusalem, Jerusalem 9190401, Israel.; ^4^ Institute of Chemistry, The Hebrew University of Jerusalem, Jerusalem 9190401, Israel.

## Abstract

The observation of spin-dependent transmission of electrons through chiral molecules has led to the discovery of chiral-induced spin selectivity (CISS). The remarkably high efficiency of the spin polarizing effect has recently gained substantial interest due to the high potential for future sustainable hybrid chiral molecule magnetic applications. However, the fundamental mechanisms underlying the chiral-induced phenomena remain to be understood fully. In this work, we explore the impact of chirality on spin angular momentum in hybrid metal/chiral molecule thin-film heterostructures. For this, we inject a pure spin current via spin pumping and investigate the spin-to-charge conversion at the hybrid chiral interface. Notably, we observe a chiral-induced unidirectionality in the conversion. Furthermore, angle-dependent measurements reveal that the spin selectivity is maximum when the spin angular momentum is aligned with the molecular chiral axis. Our findings validate the central role of spin angular momentum for the CISS effect, paving the path toward three-dimensional functionalization of hybrid molecule-metal devices via chirality.

## Teaser

Chiral molecules induce unidirectional spin-to-charge conversion with maximum spin selectivity along the molecular axis.

## INTRODUCTION

The spin-dependent charge transport through chiral molecules gives rise to a well-established phenomenon known as chiral-induced spin selectivity (CISS). Over the past decade, studies have consistently demonstrated chiral-induced spin polarization with efficiencies of up to more than 70% (*1*–*5*). Extensive investigations into the CISS effect have so far primarily focused on chiral-induced nonequilibrium spin polarization in photoemission spectroscopy and transport measurements. The initial observation of spin-polarized photoelectron emission through a polyalanine layer revealed a dependence on the chirality of the peptide molecules (*6*). Another approach to exploring the CISS effect has involved local transport measurements (*7*–*11*).

However, in addition to the CISS effect in the transport of charge carriers, it is crucial to note that chiral molecules can also substantially affect the properties of underlying metal thin films via the CISS effect resulting in an equilibrium, respectively spontaneous spin polarization. The hybrid interface between molecules and metal thin films has been a fruitful playground for engineering interfacial properties through molecular design (*12*, *13*). Hybridization between molecules and metal thin films leads to changes in the electronic and magnetic properties at the hybrid interface (*14*–*19*). Along with the widely studied effects of charge transfer and exchange coupling at hybrid interfaces, hybridization also affects the effective spin-orbit coupling (SOC) in the hybrid system. Prominent examples of the CISS effect upon hybridization is the emergence of a thermally driven spontaneous spin polarization (*18*), magnetization-dependent adsorption of chiral molecules (*10*), and manipulation of magnetization when chiral molecules are adsorbed on metallic and ferromagnetic surfaces (*17*).

Numerous experimental and theoretical efforts have tried to understand the underlying microscopic origins of the CISS effect. However, a fundamental debate persists in elucidating its microscopic mechanisms. Studies have demonstrated that SOC originating from the helical structure of chiral molecules plays a pivotal role in the CISS effect (*20*, *21*). However, the magnitude of the SOC within these molecules is often too small to account for the substantial spin-filtering effect observed in experiments. To address this challenge, several studies have focused on the role of SOC at the hybrid interface between heavy metals and chiral molecules. These studies have shown that CISS is a result of the interaction between the high SOC of the metal electrode and charge distribution at the interface of the molecule and metal (*22*–*31*). Several reports have attempted to provide a theoretical description of chiral structures by incorporating electron-phonon and electron-electron interactions. These interactions are a result of the SOC, leading to the exchange splitting of the spin channels within the structure, ultimately contributing to the phenomenon of spin filtering (*31*, *32*). On the other hand, recent studies have shown that the CISS effect can result from the chiral-induced orbital polarization effect of chiral molecules. In this framework, the topological electronic property of chiral molecules is characterized by the locking of spin and orbital angular momentum. Electrons with orbital angular momentum compatible with the molecular chirality find it easier to enter the chiral layer. This orbital polarization effect induces spin polarization mediated by the SOC in the heavy metal contact resulting in spin selectivity in the hybrid chiral molecule metal system (*33*–*35*).

While these experimental studies imply that SOC plays a crucial role, conclusive evidence has so far been lacking for a pronounced impact of the adsorption of chiral molecules on the SOC in the underlying metal thin films. In this work, we demonstrate the impact of chiral molecules on the inverse spin Hall effect (ISHE), which originates from a collection of relativistic SOC phenomena (*36*). For this, we inject a pure spin current generated by ferromagnetic resonance in a ferromagnetic insulator into a hybrid metal/chiral molecule bilayer. The SOC of the hybrid layer converts the spin current into an electromotive force via the ISHE, measurable as a voltage signal across the metal layer (*37*). The results show a chirality and spin polarization–dependent unidirectional ISHE in the hybrid chiral system confirming that SOC and spin angular momentum play a pivotal role in this CISS effect.

## RESULTS AND DISCUSSION

**Fig. 1.**
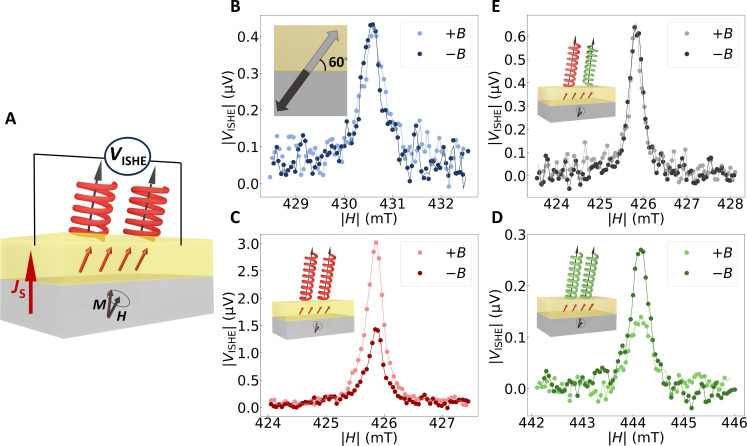
Inverse spin Hall effect voltage at ferromagnetic resonance. (**A**) Schematic representation of spin pumping at the hybrid interface between Au and chiral molecules and detection of pure spin current via the ISHE by measuring the dc voltage across the Au layer. (**B**) ISHE voltage *V*_ISHE_ measurement across ferromagnetic resonance with spin polarization at an angle of α = 60*°*. The absolute magnitude of ISHE for both positive and negative magnetic fields without molecules. (**C**) *V*_ISHE_ of the hybrid homochiral device with 36-L α-helix polyalanine molecules for both positive and negative magnetic fields. (**D**) *V*_ISHE_ of the hybrid homochiral device with 36-D α-helix polyalanine molecules for positive and negative magnetic fields. (**E**) *V*_ISHE_ for an achiral hybrid device with a racemic mixture of polyalanine molecules for positive and negative magnetic fields.

In this study, we perform spin pumping experiments in ferrimagnetic insulator (Y_3_Fe_5_O_12_, YIG, 103 nm)/heavy metal (Au, 4 nm) bilayer structures to generate a pure spin current in a nonmagnetic metal layer. The samples are placed on a grounded coplanar waveguide to excite the precession of the magnetization with an ac microwave magnetic field (10 GHz). At ferromagnetic resonance, spin angular momentum is injected from the magnetic layer into the adjacent nonmagnetic layer (*38*). Because of the ISHE, the resulting pure spin current generates an electromotive force perpendicular to the spin polarization and the spin current direction that can be detected as a voltage signal **V*_ISHE_*. The direction of the spin polarization in the pure spin current can be controlled by changing the orientation of the magnetization of the ferrimagnet using an external magnetic field (see the Supplementary Materials). A schematic illustration of the geometry of the spin pumping measurement is shown in Fig. 1A. Figure 1B shows the characteristic Lorentzian resonance shape of the ISHE voltage *V*_ISHE_ signal around the ferromagnetic resonance as a function of the applied magnetic field. Here, the magnetic field was applied at an angle of 60*°* (+*B*) and 240° (−*B*) (Fig. 1B). As expected from the symmetry of the ISHE, there is no significant difference in the absolute magnitude of *V*_ISHE_ at ferromagnetic resonance in YIG/Au between the positive (light gray) and negative (dark gray) external magnetic field. Furthermore, we note that the voltage signal *V*_ISHE_ reverses its sign when the polarity of the magnetic field is inverted (see the Supplementary Materials). However, to compare the absolute magnitude in the ISHE between positive and negative magnetic fields, the data are shown in absolute values.

### Hybrid chiral systems

To probe and quantify the impact of the CISS effect on the spin-to-charge conversion in the hybrid heavy-metal/chiral molecule system, a self-assembled monolayer of 36-L α-helix polyalanine was adsorbed on the YIG/Au sample (see schematic inset in Fig. 1C). Figure 1C shows the ISHE voltage *V*_ISHE_ of the hybrid chiral device around ferromagnetic resonance for positive (light red) and negative (dark red) magnetic fields along 60*°*. In stark contrast to the bare YIG/Au device, there is a significant change in the magnitude of *V*_ISHE_ when the polarity of the magnetic field is reversed in the hybrid chiral device. To probe the role of the chirality on the observed spin selectivity, we investigated a hybrid homochiral device with chiral molecules of the opposite handedness. Figure 1D shows also a significant difference in *V*_ISHE_ in the hybrid chiral YIG/Au/36-D α-helix polyalanine device between ferromagnetic resonance in positive (light green) and negative (dark green) magnetic fields along 60*°*. However, the sign of the difference in *V*_ISHE_ is reversed between the two opposite chiralities of the molecules. This implies that the spin selectivity in the spin-to-charge conversion efficiency for a given spin polarization direction depends on the chirality of the hybrid chiral system. Furthermore, to disentangle the effect of the chirality of the molecules from the common chirality-independent hybridization effects of molecules on metal surfaces, we have performed a control experiment on a hybrid system with a racemic mixture of the polyalanine molecules and achiral molecules (see the Supplementary Materials). The racemic mixture consists of equal fractions of both optical rotations. The ISHE voltage *V*_ISHE_ for the device with the racemic mixture is shown in Fig. 1E. Akin to the bare YIG/Au sample, the magnitude of *V*_ISHE_ at ferromagnetic resonance does not depend on the polarity of the magnetic field. Consequently, the observed asymmetry in the spin-to-charge conversion efficiency in the hybrid chiral system is directly linked to the chirality of the molecules, presenting a distinct signature of the CISS effect. The dependence of the magnitude of *V*_ISHE_ on the polarity of the spin polarization in the pure spin current implies that the chirality of the molecules substantially affects the effective SOC at the hybrid chiral interface. This result is consistent with the theoretical prediction of chiral charge transfer at the hybrid heavy metal-molecule interface resulting in a chiral charge distribution in metals (*39*).

### Angle-dependent measurements

**Fig. 2.**
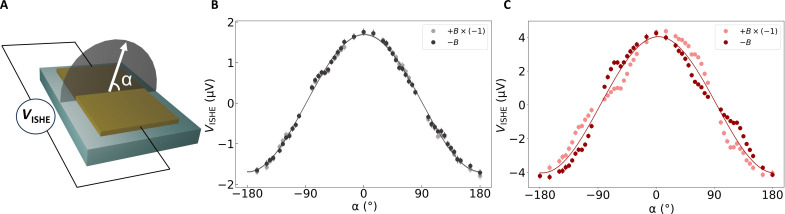
Angle dependence of the ISHE measurements. (**A**) Schematic representation of the plane of measurement where *H* represents the applied field, and α denotes the angle of the magnetic field with respect to the sample. Angle dependence of the ISHE voltage for (**B**) a bare YIG/Au device and (**C**) a hybrid chiral YIG/Au/36-L α-helix polyalanine device. For illustration purposes, the sign of the ISHE voltage signal measured with positive magnetic field has been inverted. The solid lines show the fits to the experimental data points.

In the next step, we examine the full out-of-plane angle dependence of the ISHE signal to investigate the symmetry of the spin selectivity in more detail. To maximize the detected voltage signal *V*_ISHE_, we focus on the angle dependence within the plane of rotation perpendicular to the voltage leads as shown schematically in Fig. 2A. Figure 2B shows the angle dependence of *V*_ISHE_ of the bare YIG/Au device as a function of the out-of-plane angle α of the external magnetic field for positive and negative polarity (light and dark gray, respectively). As expected from the symmetry of the ISHE, the experimental data fit well to a cos(α) function (solid line). A similar angle dependence of *V*_ISHE_ is also observed for the hybrid achiral devices (see the Supplementary Materials). In contrast to this, the angle-dependent voltage signal *V*_ISHE_ in the hybrid homochiral devices shows a significant asymmetry between the positive and negative (light and dark red, respectively) external magnetic field which reverses with opposite polarity of the magnetic field as shown in Fig. 2C for L-36 α-helix polyalanine. We emphasize that in all devices, *V*_ISHE_ vanishes around α = *±*90*°* due to the fundamental symmetry of the ISHE. The asymmetry in the angle dependence of the ISHE in homochiral systems further confirms the presence of a chiral-induced unidirectional component in the spin-to-charge conversion.

We can quantify the effective spin selectivity *S*

$$S=\frac{\left|V_{\text{ISHE}}\left(+B\right)\right|-\left|V_{\text{ISHE}}\left(-B\right)\right|}{\left|V_{\text{ISHE}}\left(+B\right)\right|+\left|V_{\text{ISHE}}\left(-B\right)\right|}$$

**Fig. 3.**
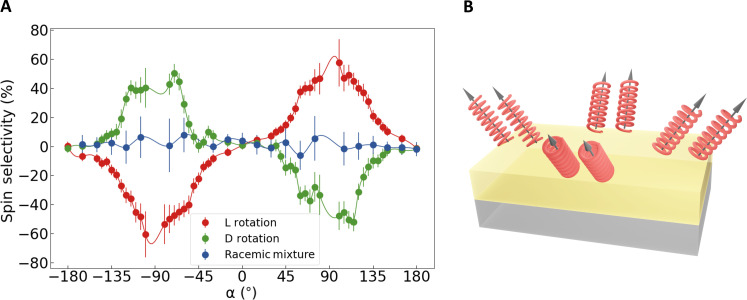
Angle dependence of the spin selectivity effect. (**A**) Spin selectivity effect represents the change in *V*_ISHE_ for different spin orientations for both positive and negative magnetic fields normalized for *V*_ISHE_ for L (red) and D (green) rotations of 36 α-helix polyalanine and the racemic mixture (blue) on Au. The solid line represents a cubic spline interpolation. (**B**) Schematic depicting the orientation of domains with different azimuthal angles of the tilted molecules on Au, where the molecules are arranged at an angle of 60*°* with respect to the sample surface.

Figure 3A shows the angle dependence of the extracted spin selectivity of the L and D rotations of the hybrid homochiral and the hybrid achiral system with the racemic mixture. The hybrid achiral system (blue) does not show any significant spin selectivity at any angle. In contrast, the L and D rotations of the hybrid homochiral systems (red and green, respectively) show a sizeable spin selectivity of up to 60%. The sign of the spin selectivity is reversed for the two opposite helicities of the L and D polyalanine molecules. We note that the spin selectivity in the homochiral devices sharply increases at *|*α*|* = 60*°* to a plateau and abruptly decreases again at *|*α*|* = 120*°*. In self-assembled monolayers, the polyalanine molecules do not arrange perfectly perpendicular to the sample surface but have been shown to tilt to approximately 60*°* with respect to the sample surface (*25*, *40*, *41*). This has been confirmed in the surface characterization of the hybrid devices investigated in this work (see the Supplementary Materials). Given this fixed out-of-plane tilt angle, the azimuthal orientation of the tilted molecules within the plane is distributed isotropically. Areas with parallel orientation of the tilted molecules form micrometer-sized domains. The ISHE measurement probes the signal averaged over many domains with different azimuthal angles as depicted schematically in Fig. 3B. As a result, the agreement between the strong and nonuniform angle dependence of the efficiency of the chiral-induced effect with the overall orientation of the molecules implies that the observed effect is vectorial. This is akin to previous reports of vectorial spin filtering via the CISS effect in charge currents (*42*).

Averaging over all homochiral devices investigated in this work, we find a mean spin selectivity of 35 ± 8% within the angle range of 60*°* < *|*α*|* < 120*°*. This magnitude of the maximum spin selectivity agrees well with previous reports of the CISS effect in charge current transport in the literature (*43*, *44*).

So far, most theoretical frameworks of the CISS effect have focused on magnetoresistance in two-terminal devices. In this geometry, no chirality-dependent magnetoresistance would be expected in the linear transport regime from fundamental symmetry considerations (*45*, *46*). In contrast, theoretical work has predicted chirality-dependent spin-to-charge conversion in a multiterminal device when a pure spin current is injected into a chiral film (*47*). Our work considers similar three-terminal devices, where a pure spin current is injected along the out-of-plane direction and the resulting ISHE voltage is detected between two transverse voltage probes. Here, the spin-to-charge conversion is modulated via a quasi-equilibrium spin polarization at the hybrid interface between the chiral molecules on the gold thin films (*18*, *28*). Furthermore, the angle dependence implies that in this experiment, the induced spin polarization direction is given by the orientation of the chiral molecules in the hybrid system rather than the external magnetic field. The experimental results confirm that chiral molecules induce chirality-dependent spin-to-charge conversion resulting in a unidirectional ISHE.

In summary, we have performed spin-pumping experiments to probe the impact of chirality on pure spin currents. Our findings show clear signatures of the CISS effect in the spin-to-charge conversion at the metal-molecule hybrid interface. In particular, we report a chiral-dependent strongly unidirectional ISHE. Through angle-dependent magnetic field measurements, we verify that the maximum spin selectivity efficiency occurs when the spin orientation aligns with the orientation of the molecular chiral axis. Thus, the chiral-induced anisotropy in the interfacial SOC acts vectorially along the axis of the chiral molecules. The CISS effect enhances and reduces the spin-to-charge conversion when the spin polarization is parallel and antiparallel to the chiral axis, respectively. These results imply that the CISS effect depends fundamentally on the electrons’ spin angular momentum and directly affects the SOC in the metal thin film. These insights open up a pathway toward targeted vectorial manipulation of hybrid spintronic devices via chirality.

## MATERIALS AND METHODS

The 36-L/D α-helix polyalanine (L/D-AHPA) [[H] CAAAAKAAAAKAAAAKAAAAKAAAAKAAAAKAAAAK-[OH]] molecules (C stands for cysteine, A for alanine, and K for lysine) were manufactured by Sigma-Aldrich. A 1 mM solution was prepared in absolute ethanol and used in the experiments. The racemic mixture was produced by mixing equal parts of the L and D solutions.

### Monolayer adsorption

Sample cleaning was performed with boiling acetone for 10 min followed by boiling isopropanol for 10 min and subsequently double distilled water. To prepare the exposed Au surface for adsorption, it was immersed in ethanol for 20 min to reduce the produced oxides. Afterward, the molecules were chemically adsorbed through their thiol head group onto the surface via a 72-hour immersion, for a dense and organized monolayer, in a 1 mM solution of the molecules in absolute ethanol in a nitrogen environment. The substrates were washed in dry ethanol and dried off. X-ray photoelectron spectroscopy (XPS) and polarization modulation-infrared reflection-adsorption spectroscopy measurements were performed to characterize the monolayer growth (see the Supplementary Materials).

### Sample fabrication process

In the fabrication of the YIG/Au sample used in this study, a 4-nm-thick layer of gold (Au) was sputter-deposited onto YIG substrates with a thickness of 103 nm, grown using the liquid phase epitaxy technique. The sputter deposition of the Au layer was carried out in an argon (Ar) plasma at a controlled rate of 0.9 Å/s with a base pressure of 5 × 10^−8^ mbar. Standard photolithography and Ar ion etching were used to fabricate the 100-μm-wide and 800-μm-long bar structures. A metallic shadow mask was used to deposit Au electrodes. Sputtering was used to deposit 5 nm of Cr and 50 nm of Au electrodes.

### Measurement procedure

The YIG/Au sample was mounted on top of a strip line of the grounded coplanar waveguide. The input microwave power remained consistently fixed at 10 dBm, and the microwave frequency was set to 10 GHz. The ferromagnetic resonance and ISHE measurements were carried out using a lock-in technique modulating the amplitude of the microwave signal at a modulation frequency of 1.5 kHz. We have verified that the amplitude of the microwave absorption at ferromagnetic resonance is comparable for both polarities of the magnetic field (see the Supplementary Materials). The experiments have been performed on two samples each for both enantiomers and the racemic mixture. The angle-dependent ISHE voltage measurements have been performed on all devices before the adsorption of molecules to verify that the ISHE response is fully antisymmetric between the positive and negative polarity of the external magnetic field as previously reported and expected for YIG/Au bilayer samples. After the adsorption of the molecules on the YIG/Au devices, the full angle dependence of the ISHE voltage measurement was repeated.

## Supplementary Materials

### This PDF file includes:

Supplementary Text Figs. S1 to S13 Tables S1 to S3 References
