# Supplementary material for: Chiral-induced unidirectional spin-to-charge conversion
Source: Sci Adv. 2025 Jan 1;11(1):eado4285. doi: 10.1126/sciadv.ado4285 (PMC11691638; doi:10.1126/sciadv.ado4285)
Supplement: Supplementary file 1 — Supplementary Text Figs. S1 to S13 Tables S1 to S3 References [file sciadv.ado4285_sm.pdf]

Science Advances
AAAS

Supplementary Materials for

Chiral-induced unidirectional spin-to-charge conversion

Ashish Moharana *et al.*

Corresponding author: Angela Wittmann, a.wittmann@uni-mainz.de

Sci. Adv. **11**, eado4285 (2025)
DOI: 10.1126/sciadv.ado4285

This PDF file includes:

Supplementary Text
Figs. S1 to S13
Tables S1 to S3
References

L α-helix polyalanine monolayer characterization

Figure S1 shows the results of the X-ray photoelectron spectroscopy (XPS) characterization of the L α-helix polyalanine monolayer adsorbed on Au with a thiol end group. We have quantified the elemental concentrations from the XPS results on the hybrid homochiral sample (Table S1) and a bare gold substrate (Table S2). The increased concentrations of O, C, and N in the L α-helix polyalanine sample, in contrast to the bare gold substrate, signify the appearance of polyalanine molecules. The presence of S indicates the attachment of thiol-functionalized end groups, confirming the monolayer formation on the gold substrate.

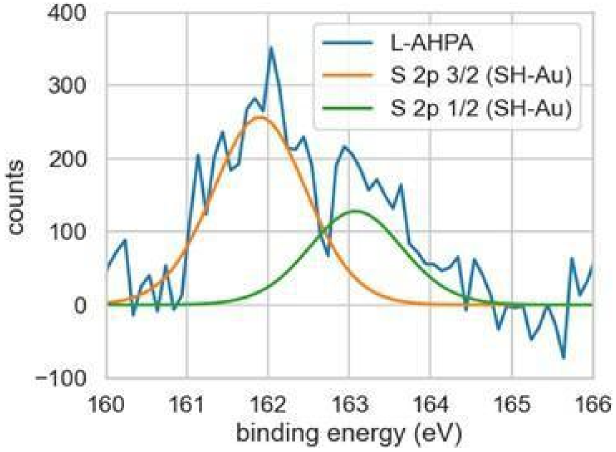

Fig. S1. X-ray photoelectron spectroscopy (XPS) characterization of an L *α*-helix polyalanine monolayer adsorbed on Au with a thiol end group.

Table S1. Quantification of X-ray photoelectron spectroscopy (XPS) results for elemental composition of an L α-helix polyalanine monolayer on a gold substrate.

	Atomic conc. [%]	Error [%]	Mass conc. [%]	Error [%]
S 2p	1.29	0.32	0.77	0.19
N 1s	5.81	0.31	1.51	0.08
C 1s	51.48	0.62	11.47	0.22
O 1s	19.39	0.38	5.76	0.11
Au 4f	22.02	0.28	80.49	0.28

Table S2. Quantification of X-ray photoelectron spectroscopy (XPS) results for elemental composition for a bare gold substrate.

	Atomic conc. [%]	Error [%]	Mass conc. [%]	Error [%]
S 2p	0		0	
N 1s	1.1	0.26	0.15	0.03
C 1s	41.04	0.5	4.73	0.09
O 1s	8.14	0.3	1.25	0.05
Au 4f	49.72	0.45	93.88	0.1

The increased concentrations of O, C, and N in the α-helix polyalanine monolayer sample, in contrast to the clean substrate, signify the appearance of polyalanine molecules. The presence of S indicates the attachment of thiol-functionalized end groups, confirming the monolayer formation on the gold substrate.

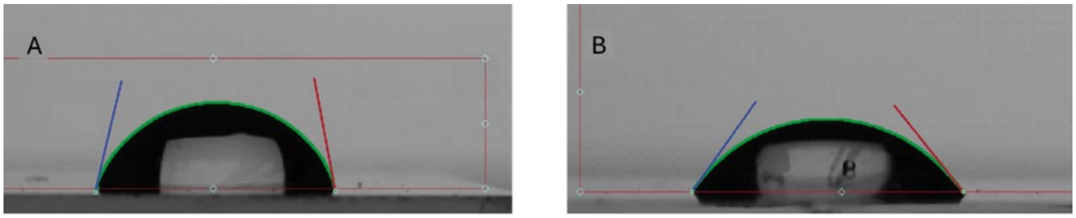

Fig. S2: Contact angle characterization of L α-helix polyalanine monolayer adsorbed on Au through thiol end group. (A) after cleaning YIG/Au surface (B) after adsorption on L-α helix polyalanine.

The figure displays a water droplet on the YIG/Au surface (A) after cleaning and (B) after adsorption on L-α helix polyalanine. Measurement was done using the commercial Ossila Contact Angle Goniometer. The angle between the surface and the droplet characterizes the wettability of a surface by water. Three droplets were measured before and after adsorption and the average angle was obtained. The full data is described in supplementary table 2. Before adsorption (A), the contact angle is 75° ± 2°. After adsorption (B), the contact angle decreases to 53° ± 1°, suggesting increased wetting due to the adsorbed monolayer.

Table S3: Contact Angle Characterization of L α-helix polyalanine monolayer adsorbed on Au through thiol end group. Contact angle data was obtained from four droplets in different areas of the sample before (top-purple) and after (bottom-blue) adsorption of the chiral molecules.

		Left Angle (°)	Right Angle (°)	Average Angle (°)	Left RMSE	Right RMSE	Droplet Width (Pixels)
**Clean**	0	77.25	76.98	77.11	2.09	0.82	346.6
	1	74.22	71.24	72.73	0.44	0.45	445
	2	65.59	74.93	70.26	0.47	0.38	433.3
	3	76.5	79.4	77.95	0.53	0.35	424.1
	**Average**	**74.5 ± 1.5**					
		**Left Angle (°)**	**Right Angle (°)**	**Average Angle (°)**	**Left RMSE**	**Right RMSE**	**Droplet Width (Pixels)**
**L-AHPA**	0	47.66	49.55	48.6	0.60	0.69	535.2
	1	54.44	51.2	52.82	0.51	0.58	497.6
	2	56.04	55.13	55.58	0.59	0.58	500
	3	55.89	57.41	56.65	1.29	0.56	485.5
	**Average**	**53.4 ± 1.2**					

Polarization modulation-infrared reflection-adsorption spectroscopy

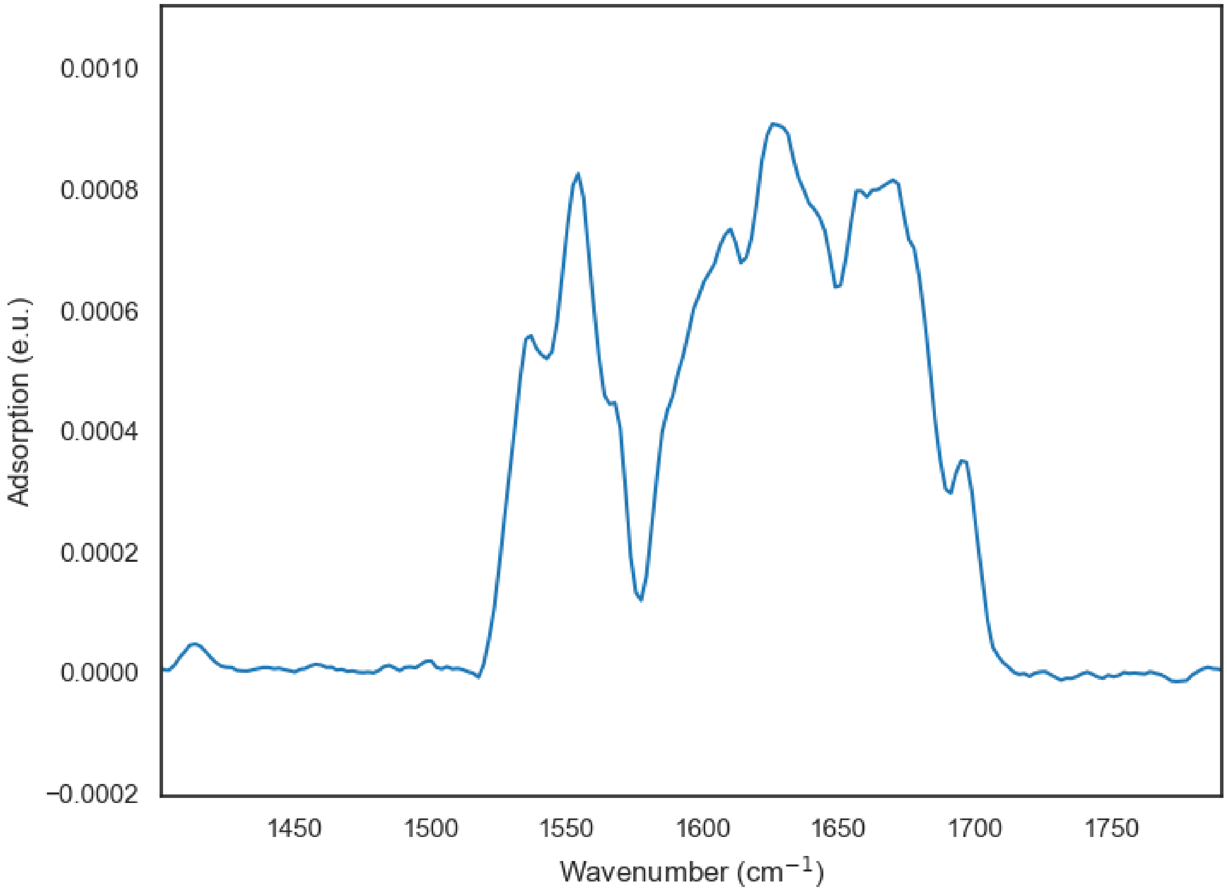

Fig. S3: PM-IRRAS spectra of L-polyalanine monolayer on a gold surface.
The structure and tilt angle of the chiral monolayer on the YIG/Au(4nm) sample was characterized by Polarization modulation-infrared reflection-adsorption spectroscopy (PM-IRRAS). Measurements were conducted at room temperature under N2 atmosphere in a reflection-absorption cell (Harrick, Inc.) with a PM-FTIR spectrometer (PMA-50 coupled to Vertex 70, Bruker).

The energies of amide-I and amide-II vibrations, 1663 and 1549 cm−1 respectively, indicate that the monolayer is in a helix form. From their ratio, the tilt angle of the molecules' adsorption relative to the surface can be obtained.
The following formula was used to obtain the tilt angle, similar to previous works (48-50):

$$\frac{I_1}{I_2} = 1.5 * \left(\frac{(3\gamma\ - 1)(3\theta_1\ - 1) + 2}{(3\gamma\ - 1)(3\theta_2\ - 1) + 2}\right)$$

where I1 and I2 are the absorbance of amide I and II bands (1663 and 1549 cm−1 respectively), γ is the tilt angle of the helical axis with respect to the surface, and θ1,2 are the angles between the transition moment and the helix axis. The values of θ1 and θ2 were set to 39° and 75°, respectively (51). Using this, a tilt angle of 61° of the molecules with respect to the surface was calculated.

Microwave absorption and ISHE voltage measurements

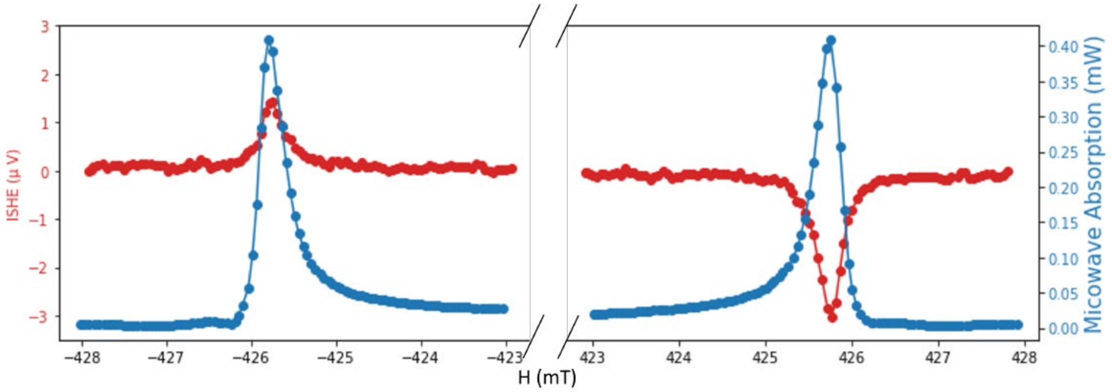

Fig. S4. FMR Spectrum of a hybrid chiral device with L α-helix polyalanine. Inverse spin Hall effect voltage V_{ISHE} (red) and the microwave absorption (blue) at ferromagnetic resonance in an external field at 60° for a hybrid homochiral YIG/Au/chiral molecules sample with L α-helix polyalanine. While the absorption is comparable in positive and negative fields, the inverse spin Hall effect signal shows a significant difference depending on the polarity of the external field.

Angle dependence of the magnetization

To verify that the magnetization is parallel to the external field at all angles, we have extracted the angle ϕ of the magnetization as a function of the angle α of the applied field. The magnetization precession can be described by the Landau Lifshitz Gilbert equation (52)

$$\frac{f}{\gamma} = [H_{\mathrm{FMR}} \cos(\varphi - \theta) + \mu_0 M_s \cos(2\varphi)] \times H_{\mathrm{FMR}} \cos(\varphi - \theta) - \mu_0 M_s \sin^2(\varphi),$$

where f denotes the microwave frequency, γ the gyromagnetic ratio, H_{FMR} the resonance field, and M_s the saturation magnetization. Using this to fit the experimental data (Fig. S10a), reveals a linear dependence of the angle of the magnetization on the angle of the magnetic field (Fig. S10b) and, thus, confirms that the resonance field overcomes the anisotropy of the ferromagnetic insulator.

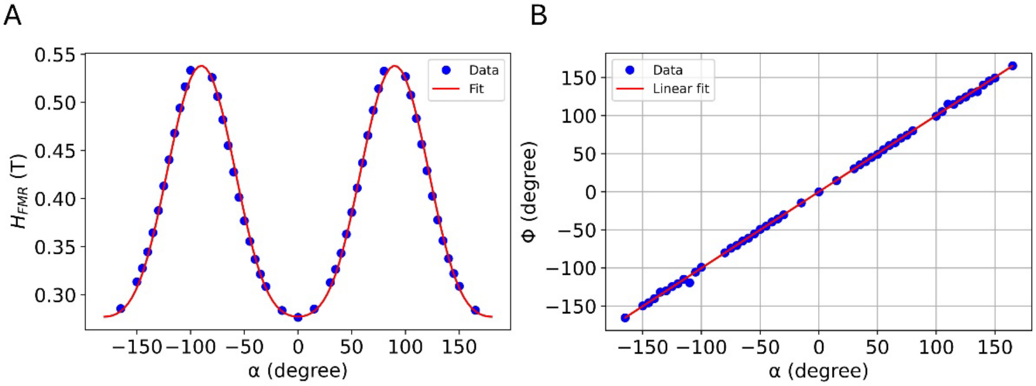

Fig. S5. Angle dependence of the magnetization. (a) Measured angular dependence of the magnetic resonance field (blue circles). The red line shows the numerical solution of

the Landau-Lifshitz-Gilbert equation. (b) Angle dependence of the extracted magnetization angle (ϕ) as a function of the magnetic field angle (α).

ISHE measurements for pristine YIG/Au

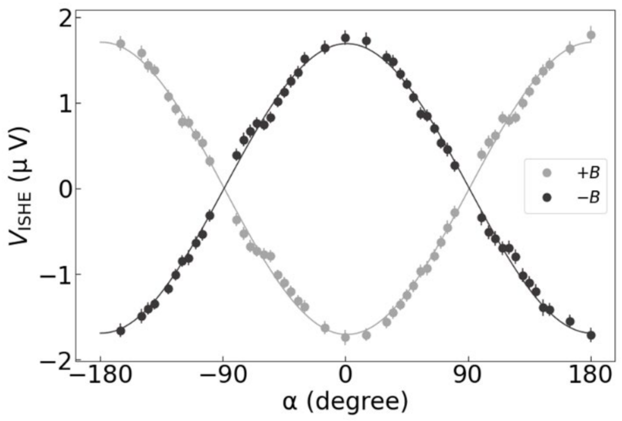

Fig. S6. Angle dependence of the ISHE voltage for a bare YIG/Au device.

Additional ISHE measurements for hybrid devices with polyalanine molecules

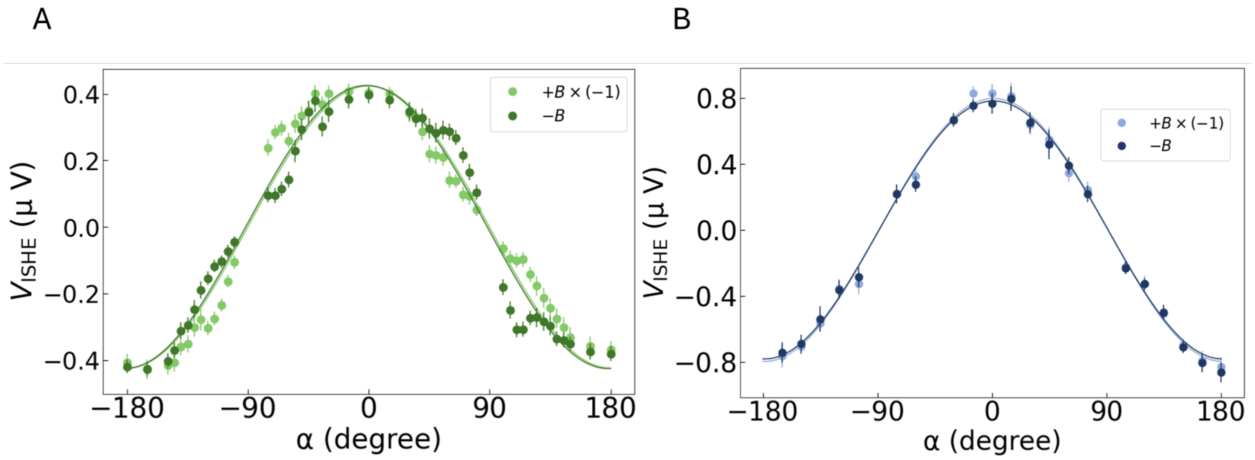

Fig. S7: Angle dependence of the ISHE voltage signal for hybrid devices. Voltage signal for (A) a hybrid chiral YIG/Au/36 - D α-helix polyalanine device and (B) a racemic mixture of α-helix polyalanine device. For illustration purposes, the sign of the ISHE voltage signal measured in positive magnetic field has been inverted. The solid lines show fits of a cos(α) function to the experimental data points.

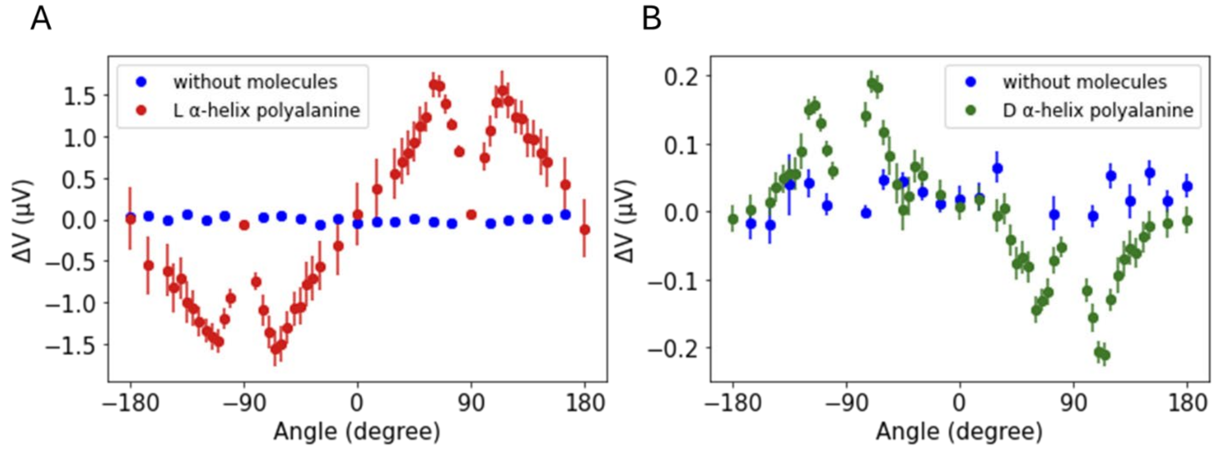

Fig. S8: Angle dependence measurement for the absolute change in the V_{ISHE} for positive and negative fields. Hybrid devices with (A) homochiral 36 - L α-helix polyalanine (red) and (B) 36 - D α-helix polyalanine (red). The blue points show the data for the bare YIG/Au devices.

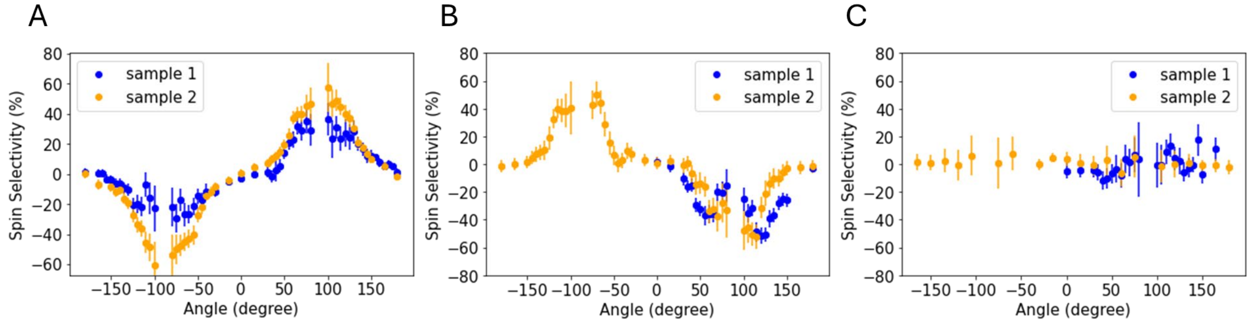

Fig. S9: Reproducibility of angle-dependent spin selectivity measurements on different devices. Spin selectivity effect for Sample 1 and 2 for (A) 36 - L α-helix polyalanine, (B) 36 - D α-helix polyalanine, (C) racemic Mixture.

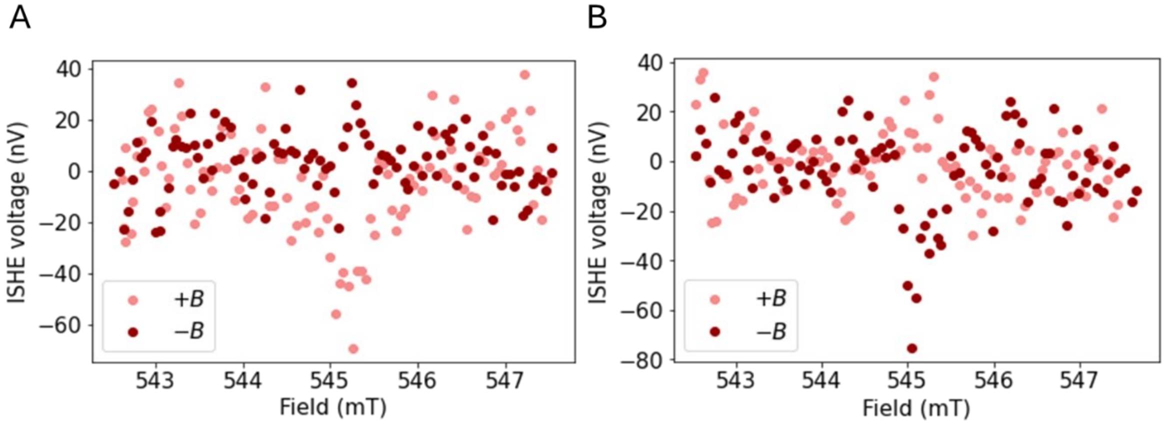

Fig. S10: FMR spectra of a hybrid homochiral device in out-of-plane magnetic field. V_{ISHE} amplitude at (A) α = +90° and (B) α = -90° for positive and negative polarity of the magnetic field for a hybrid homochiral YIG/Au/36 - L α-helix polyalanine device.

Additional ISHE measurements for a hybrid device with achiral molecules

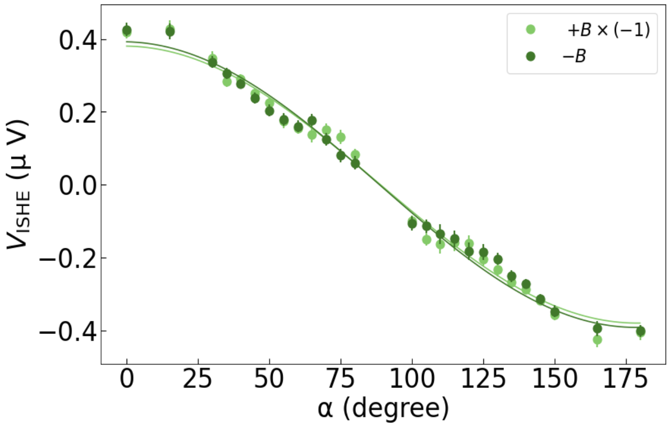

Fig. S11: ISHE measurement for a hybrid device with achiral molecules. Angle dependence of the V_{ISHE} signal for positive and negative field for achiral molecules (11- mercaptoundecanoic acid).

Simple model for spin selectivity of different molecule orientations

To illustrate that the in-plane components of the different domains of the tilted molecules enhance each other, we have put together a simple model. All molecules are oriented at a Φ = 60° angle with respect to the sample surface. With this given out-of-plane angle Φ, the angle θ in the plane of the surface is distributed isotropically across the sample in different domains.

We start with the straightforward case of all molecules oriented along one axis (Φ = 60° and θ = 0°). Presuming that the spin selectivity is given by the component of the spin polarization along the chiral axis, we can approximate the resulting spin selectivity with a simple cos(α - 60°)

function. The resulting spin selectivity has a maximum at α = 60° and a minimum at α = -120° as shown in Fig. S12 (blue line). We can now add the contributions from the opposite side of the cone but still within the plane of rotation of the spin polarization (Φ = 60° and θ = 180°). This can be described with a cos(α - 120°) function (orange line). Any contributions from other molecules within the cone will increase the magnitude of the spin selectivity within each of the hemispheres. Thus, both the in-plane and the out-of-plane contributions are additive within the respective hemispheres.

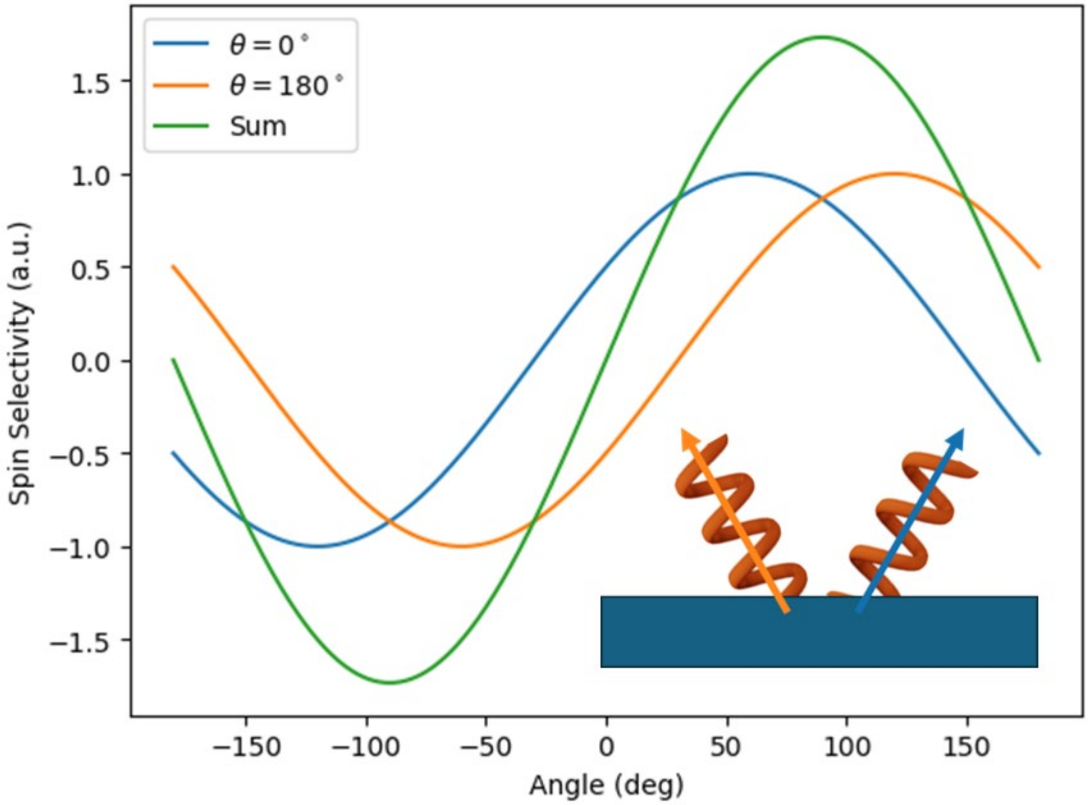

Fig. S12: Angle dependence of the spin selectivity modeled using a trigonometric cosine function. The addition of the spin-selectivity from two domains with the azimuthal angles θ = 0° (blue) and θ = 0° (orange) results in a trigonometric function with a maximum at α = 90° and minimum at α = -90° (green).

To illustrate that this also holds true if the spin selectivity is more sensitive to the relative angle between the chiral axis and the injected spin polarization direction, we can modify this simple model by expressing the angle dependence of the spin selectivity with a Gaussian function. For molecules along Φ = 60° and θ = 0°, we can express this by exp(-((α - 60°)/w)^2)-exp(-((α + 120°)/w)^2). The resulting angle dependence is shown in Fig. S13 (dark blue line) for a width of w = 20°. Similarly to the model based on the trigonometric description of the spin selectivity above, all contributions from molecules with other in-plane angles increase the sum (bold red line) for angles within the cone of the molecules.

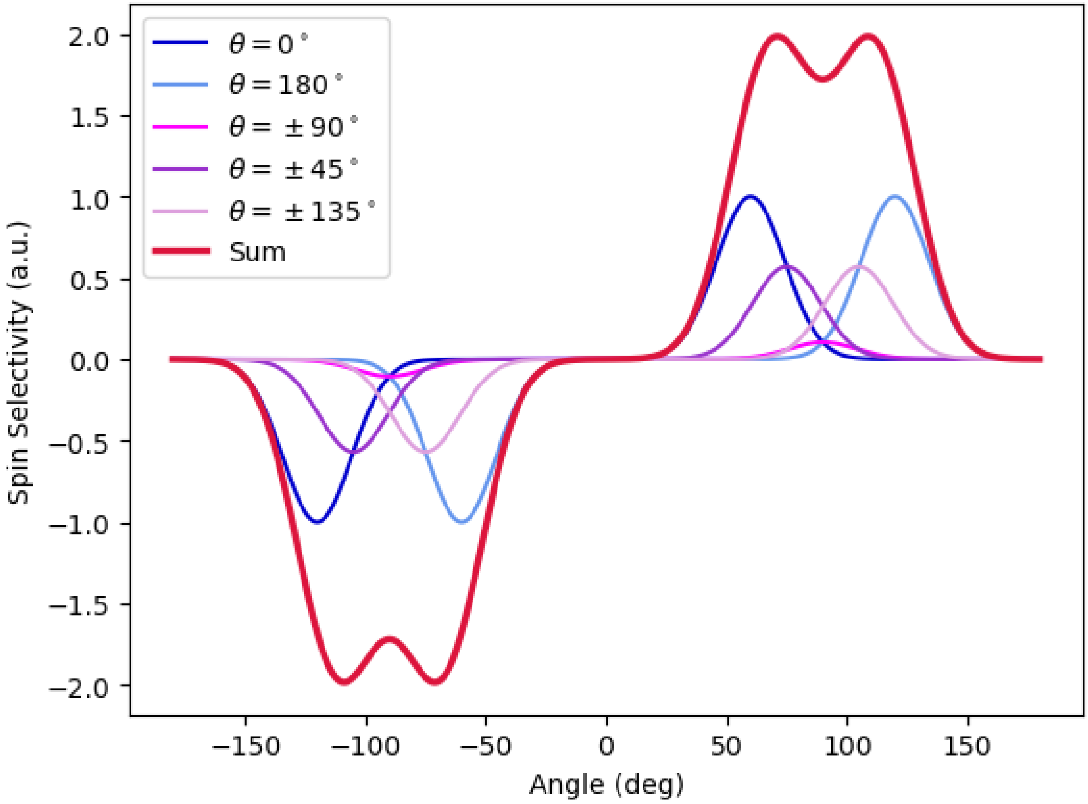

Fig. S13: Angle dependence of the spin selectivity modeled based on Gaussian functions.

REFERENCES AND NOTES

1. R. Naaman, D. H. Waldeck, Spintronics and chirality: Spin selectivity in electron transport through chiral molecules. *Annu. Rev. Phys. Chem.* **66**, 263–281 (2015).

2. R. Naaman, Y. Paltiel, D. H. Waldeck, Chiral molecules and the electron spin. *Nat. Rev. Chem.* **3**, 250–260 (2019).

3. R. Naaman, Y. Paltiel, D. H. Waldeck, Chiral molecules and the spin selectivity effect. *J. Phys. Chem. Lett.* **11**, 3660–3666 (2020).

4. R. Naaman, D. H. Waldeck, Chiral-induced spin selectivity effect. *J. Phys. Chem. Lett.* **3**, 2178–2187 (2012).

5. H. Al-Bustami, S. Khaldi, O. Shoseyov, S. Yochelis, K. Killi, I. Berg, E. Gross, Y. Paltiel, R. Yerushalmi, Atomic and molecular layer deposition of chiral thin films showing up to 99% spin selective transport. *Nano Lett.* **22**, 5022–5028 (2022).

6. B. Göhler, V. Hamelbeck, T. Z. Markus, M. Kettner, G. F. Hanne, Z. Vager, R. Naaman, H. Zacharias, Spin selectivity in electron transmission through self-assembled monolayers of double-stranded DNA. *Science* **331**, 894–897 (2011).

7. H. Lu, J. Wang, C. Xiao, X. Pan, X. Chen, R. Brunecky, J. J. Berry, K. Zhu, M. C. Beard, Z. V. Vardeny, Spin-dependent charge transport through 2D chiral hybrid lead-iodide perovskites. *Sci. Adv.* **5**, eaay0571 (2019).

8. E. Z. Smolinsky, A. Neubauer, A. Kumar, S. Yochelis, E. Capua, R. Carmieli, Y. Paltiel, R. Naaman, K. Michaeli, Electric field-controlled magnetization in GaAs/AlGaAs heterostructures–chiral organic molecules hybrids. *J. Phys. Chem. Lett.* **10**, 1139–1145 (2019).

9. S. Ghosh, S. Mishra, E. Avigad, B. P. Bloom, L. T. Baczewski, S. Yochelis, Y. Paltiel, R. Naaman, D. H. Waldeck, Effect of chiral molecules on the electron's spin wavefunction at interfaces. *J. Phys. Chem. Lett.***11**, 1550–1557 (2020)

10. M. R. Safari, F. Matthes, K. H. Ernst, D. E. Bürgler, C. M. Schneider, Deposition of chiral heptahelicene molecules on ferromagnetic Co and Fe thin-film substrates. *Nanomaterials* **12**, 3281 (2022).

11. R. Sun, Z. Wang, B. P. Bloom, A. H. Comstock, C. Yang, A. McConnell, C. Clever, M. Molitoris, D. Lamont, Z.-H. Cheng, Z. Yuan, W. Zhang, A. Hoffmann, J. Liu, D. H. Waldeck, D. Sun, Colossal anisotropic absorption of spin currents induced by chirality. *Sci. Adv.* **10**, eadn3240 (2024).

12. M. Cinchetti, V. A. Dediu, L. E. Hueso, Activating the molecular spinterface. *Nat. Mater.* **16**, 507–515 (2017).

13. A. Wittmann, G. Schweicher, K. Broch, J. Novak, V. Lami, D. Cornil, E. R. McNellis, O. Zadvorna, D. Venkateshvaran, K. Takimiya, Y. H. Geerts, J. Cornil, Y. Vaynzof, J. Sinova, S. Watanabe, H. Sirringhaus, Tuning spin current injection at ferromagnet-nonmagnet interfaces by molecular design. *Phys. Rev. Lett.* **124**, 027204 (2020).

14. J. Brede, N. Atodiresei, S. Kuck, P. Lazić, V. Caciuc, Y. Morikawa, G. Hoffmann, S. Blügel, R. Wiesendanger, Spin-and energy-dependent tunneling through a single molecule with intramolecular spatial resolution. *Phys. Rev. Lett.* **105**, 047204 (2010).

15. N. Atodiresei, J. Brede, P. Lazić, V. Caciuc, G. Hoffmann, R. Wiesendanger, S. Blügel, Design of the local spin polarization at the organic-ferromagnetic interface. *Phys. Rev. Lett.* **105**, 066601 (2010).

16. C. F. Hermanns, K. Tarafder, M. Bernien, A. Krüger, Y. M. Chang, P. M. Oppeneer, W. Kuch, Magnetic coupling of porphyrin molecules through graphene. *Adv.Mater.* **25**, 3473–3477 (2013).

17. O. Ben Dor, S. Yochelis, A. Radko, K. Vankayala, E. Capua, A. Capua, S. H. Yang, L. T. Baczewski, S. S. Parkin, R. Naaman, Y. Paltiel, Magnetization switching in ferromagnets by adsorbed chiral molecules without current or external magnetic field. *Nat. Commun.* **8**, 14567 (2017).

18. K. Kondou, M. Shiga, S. Sakamoto, H. Inuzuka, A. Nihonyanagi, F. Araoka, M. Kobayashi, S. Miwa, D. Miyajima, Y. Otani. Chirality-induced magnetoresistance due to thermally driven spin polarization. *J. Am. Chem. Soc.*, **144**, 7302–7307 (2022).

19. M. Ozeri, J. Xu, G. Bauer, L. A. Olthof Olde, G. Kimbell, A. Wittmann, S. Yochelis, J. Fransson, J. W. Robinson, Y. Paltiel, O. Millo. Modification of weak localization in metallic thin films due to the adsorption of chiral molecules. *J. Phys. Chem. Lett.* **14**, 4941–4948 (2023).

20. A. M. Guo, Q. F. Sun, Spin-selective transport of electrons in DNA double helix. *Phys. Rev. Lett.* **108**, 218102 (2012).

21. R. Gutierrez, E. Díaz, R. Naaman, G. Cuniberti, Spin-selective transport through helical molecular systems. *Phys. Rev. B* **85**, 081404 (2012).

22. G. Contini, S. Turchini, S. Sanna, D. Catone, J. Fujii, I. Vobornik, T. Prosperi, N. Zema, Transfer of chirality from adsorbed chiral molecules to the substrate highlighted by circular dichroism in angle-resolved valence photoelectron spectroscopy. *Phys. Rev. B* **86**, 035426 (2012).

23. O. Ben Dor, N. Morali, S. Yochelis, L. T. Baczewski, Y. Paltiel, Local light-induced magnetization using nanodots and chiral molecules. *Nano Lett.* **14**, 6042–6049 (2014).

24. B. P. Bloom, B. M. Graff, S. Ghosh, D. N. Beratan, D. H. Waldeck, Chirality control of electron transfer in quantum dot assemblies. *J. Am. Chem. Soc.* **139**, 9038–9043 (2017).

25. N. T. Ha, A. Sharma, D. Slawig, S. Yochelis, Y. Paltiel, D. R. Zahn, G. Salvan, C. Tegenkamp, Charge-ordered α-helical polypeptide monolayers on Au (111). *J. Phys. Chem. C* **124**, 5734–5739 (2020).

26. S. Alwan, Y. Dubi, Spinterface origin for the chirality-induced spin-selectivity effect. *J. Am. Chem. Soc.* **143**, 14235–14241 (2021).

27. Y. Dubi, Spinterface chirality-induced spin selectivity effect in bio-molecules. *Chem. Sci.* **13**, 10878–10883 (2022).

28. J. Fransson, Charge redistribution and spin polarization driven by correlation induced electron exchange in chiral molecules. *Nano Lett.* **21**, 3026–3032 (2021).

29. S. Naskar, V. Mujica, C. Herrmann, Chiral-induced spin selectivity and non-equilibrium spin accumulation in molecules and interfaces: A first-principles study. *J. Phys. Chem. Lett.* **14**, 694–701 (2023).

30. P. Hedegård, Spin dynamics and chirality induced spin selectivity. *J. Chem. Phys.* **159**, 104104 (2023).

31. J. Fransson, Vibrational origin of exchange splitting and chiral-induced spin selectivity. *Phys. Rev. B* **102**, 235416 (2020).

32. J. Fransson, Chirality-induced spin selectivity: The role of electron correlations. *J. Phys. Chem. Lett.* **10**, 7126–7132 (2019).

33. J. Gersten, K. Kaasbjerg, A. Nitzan, Induced spin filtering in electron transmission through chiral molecular layers adsorbed on metals with strong spin-orbit coupling. *J. Chem. Phys.* **139**, 114111 (2013).

34. Y. Liu, J. Xiao, J. Koo, B. Yan, Chirality-driven topological electronic structure of DNA-like materials. *Nat. Mater.* **20**, 638–644 (2021).

35. Y. Adhikari, T. Liu, H. Wang, Z. Hua, H. Liu, E. Lochner, P. Schlottmann, B. Yan, J. Zhao, P. Xiong, Interplay of structural chirality, electron spin and topological orbital in chiral molecular spin valves. *Nat. Commun.* **14**, 5163 (2023).

36. J. Sinova, D. Culcer, Q. Niu, N. A. Sinitsyn, T. Jungwirth, A. H. MacDonald, Universal intrinsic spin Hall effect. *Phys. Rev. Lett.* **92**, 126603 (2004).

37. J. Sinova, S. O. Valenzuela, J. Wunderlich, C. H. Back, T. Jungwirth, Spin Hall effects. *Rev. Mod. Phys.* **87**, 1213 (2015).

38. E. Saitoh, M. Ueda, H. Miyajima, G. Tatara, Conversion of spin current into charge current at room temperature: Inverse spin-Hall effect. *Appl. Phys. Lett.* **88**, 182509 (2006).

39. M. Du, X. Liu, X. Liu, S. Xie, Chirality transfer induced spin selectivity effect in a molecule-metal heterojunction. *Phys. Rev. B* **108**, 125419 (2023).

40. T. Sakurai, S. Oka, A. Kubo, K. Nishiyama, I. Taniguchi, Formation of oriented polypeptides on Au(111) surface depends on the secondary structure controlled by peptide length. *J. Pept. Sci.* **12**, 396–402 (2006).

41. N. Sukenik, F. Tassinari, S. Yochelis, O. Millo, L. T. Baczewski, Y. Paltiel, Correlation between ferromagnetic layer easy axis and the tilt angle of self assembled chiral molecules. *Molecules* **25**, 6036 (2020).

42. C. Badala Viswanatha, J. Stöckl, *B. Arnoldi*, S. Becker, M. Aeschlimann, B. Stadtmüller, Vectorial electron spin filtering by an all-chiral metal–molecule heterostructure. *J. Phys. Chem. Lett.* **13**, 6244–6249 (2022).

43. V. Heß, R. Friedrich, F. Matthes, V. Caciuc, N. Atodiresei, D. E. Bürgler, S. Blügel, C. M. Schneider, Magnetic subunits within a single molecule–surface hybrid. *New J. Phys.* **19**, 053016 (2017).

44. M. R. Safari, F. Matthes, C. M. Schneider, K. H. Ernst, D. E. Bürgler, Spin-selective electron transport through single chiral molecules. *Small* **20**, 2308233 (2023).

45. X. Yang, C. H. Van Der Wal, B. J. Van Wees, Spin-dependent electron transmission model for chiral molecules in mesoscopic devices. *Phys. Rev. B* **99**, 024418 (2019).

46. X. Yang, C. H. Van Der Wal, B. J. Van Wees, Detecting chirality in two-terminal electronic nanodevices. *Nano Lett.* **20**, 6148–6154 (2020).

47. X. Yang, B. J. van Wees, Linear-response magnetoresistance effects in chiral systems. *Phys. Rev. B* **104**, 155420 (2021).

48. N. Peer, I. Dujovne, S. Yochelis, Y. Paltiel, Nanoscale charge separation using chiral molecules. *ACS Photonics* **2**, 1476–1481 (2015).

49. D. E. López-Pérez, G. Revilla-López, D. Jacquemin, D. Zanuy, B. Palys, S. Sek, C. Alemán, Intermolecular interactions in electron transfer through stretched helical peptides. *Phys. Chem. Chem. Phys.* **14**, 10332–10344 (2012).

50. R. Naaman, Z. Vager, "Spin selective electron transmission through monolayers of chiral molecules" in *Electronic and Magnetic Properties of Chiral Molecules and Supramolecular Architectures*, R. Naaman, D. N. Beratan, D. Waldeck, Eds. (Springer, 2011) pp. 237.

51. Y. Miura, S. Kimura, Y. Imanishi, J. Umemura, Formation of oriented helical peptide layers on a gold surface due to the self-assembling properties of peptides. *Langmuir* **14**, 6935–6940 (1998).

52. K. Ando, S. Takahashi, J. Ieda, Y. Kajiwara, H. Nakayama, T. Yoshino, K. Harii, Y. Fujikawa, M. Matsuo, S. Maekawa, E. Saitoh, Inverse spin-Hall effect induced by spin pumping in metallic system, *J. Appl. Phys.* **109**, 103913 (2011).
